# Detection of extraprostatic extension by transperineal multiparametric magnetic resonance imaging-ultrasound fusion targeted combined with systemic template prostate biopsy

**DOI:** 10.1186/s13000-023-01386-w

**Published:** 2023-09-11

**Authors:** Hao-Wen Chuang, Shulin Wu, Sharron X. Lin, Ting Zhao, Michelle M. Kim, Mukesh Harisinghani, Adam S. Feldman, Douglas M. Dahl, Chin-Lee Wu

**Affiliations:** 1 Department of Pathology, Massachusetts General Hospital, Harvard Medical School, Boston, MA USA; 2 Department of Pathology and Laboratory Medicine, Kaohsiung Veterans General Hospital, Kaohsiung, TW Taiwan; 3 Institute of Oral Biology, School of Dentistry, National Yang Ming Chiao Tung University, Taipei, TW Taiwan; 4 Department of Urology, Massachusetts General Hospital, Harvard Medical School, Boston, MA USA; 5 Department of Radiology, Massachusetts General Hospital, Harvard Medical School, Boston, MA USA

**Keywords:** Extraprostatic extension, Prostate cancer, Transperineal, Transrectal, Fusion biopsy, Template

## Abstract

**Background:**

Extraprostatic extension (EPE) of prostate cancer (PCa) on transrectal (TR) needle core biopsy (Bx) is a rare histopathological finding that can help in clinical decision-making. The detection efficiency of the transperineal (TP) approach is yet to be explored.

**Methods:**

We retrospectively reviewed 2848 PCa cases using concomitant systemic template biopsy (SBx) and multiparametric magnetic resonance imaging (MRI)-ultrasound fusion-targeted biopsy (TBx) using the TR (n = 1917) or TP (n = 931) approach at our institution between January 2015 and July 2022. We assessed and compared clinical, MRI, and biopsy characteristics using different approaches (TP and TR) and methods (SBx and TBx).

**Results:**

In total, 40 EPE cases were identified (40/2848, 1.4%). TP showed a significantly higher EPE detection rate compared to TR in SBx (TR:0.7% vs. TP:1.6%; *p* = 0.028) and TBx (TR:0.5% vs. TP:1.2%; *p* = 0.033), as well as the combined methods (2.1% vs. 1.1%, *p* = 0.019). A significantly higher incidence of EPEs was found at non-base sites in TP than in TR (76.7% vs. 50%, *p* = 0.038). SBx showed a higher EPE detection rate than TBx; however, the difference was not statistically significant. TP showed higher prostate-specific antigen density (0.35 vs. 0.17, *p* = 0.005), higher frequency of GG4-5 in the cores with EPE (65.0% vs. 50.0%, *p* = 0.020), and more PCa-positive SBx cores (10 vs. 8, *p* = 0.023) compared to the TR.

**Conclusions:**

TP may improve EPE detection compared with TR and should be applied to patients with adverse pre-biopsy features.

**Supplementary Information:**

The online version contains supplementary material available at 10.1186/s13000-023-01386-w.

## Background

Extraprostatic extension (EPE) of prostate cancer (PCa) is a common pathological finding in radical prostatectomy (RP), with a frequency ranging from 23 to 67% in previous studies. In addition, it is a well-established prognostic indicator of the biochemical recurrence (BCR) [1–3]. EPE can also be detected using prostate needle biopsy; however, the prevalence is very low (0.19–1.37%) according to three large series [2, 4, 5]. The role of multiparametric magnetic resonance imaging (mpMRI) in detecting PCa EPE has been investigated previously. In a recent meta-analysis summarizing data from 75 mpMRI studies, the pooled sensitivity of mpMRI for EPE detection was 57% [6]. Furthermore, mpMRI-ultrasound (US) fusion-targeted biopsy (TBx) has been reported to improve the detection of clinically significant (cs) PCa (csPCa) compared with systematic template biopsy (SBx) [7, 8]. Recently, Baumgartner et al. [9] reported an EPE detection rate of 1.5% using TBx in a small cohort (5/333). Four of the five identified EPE cases underwent concurrent SBx and TBx; three cases were identified as EPE only by TBx, and one case was identified as EPE only by SBx. Their results indicated that TBx outperformed SBx in the identification of EPE. The transperineal (TP) biopsy approach has gained popularity owing to its higher detection rate of PCa, improved detection of anterior cancer, lower risk of complications, and feasibility in the outpatient setting under local anesthesia [10]. However, the efficiency of EPE detection using the TP approach is yet to be studied. This study aimed to compare the EPE frequency detected using different approaches (TP and transrectal [TR]), as well as different methods (SBx and TBx).

## Materials and methods

### Study population

**Fig. 1:**
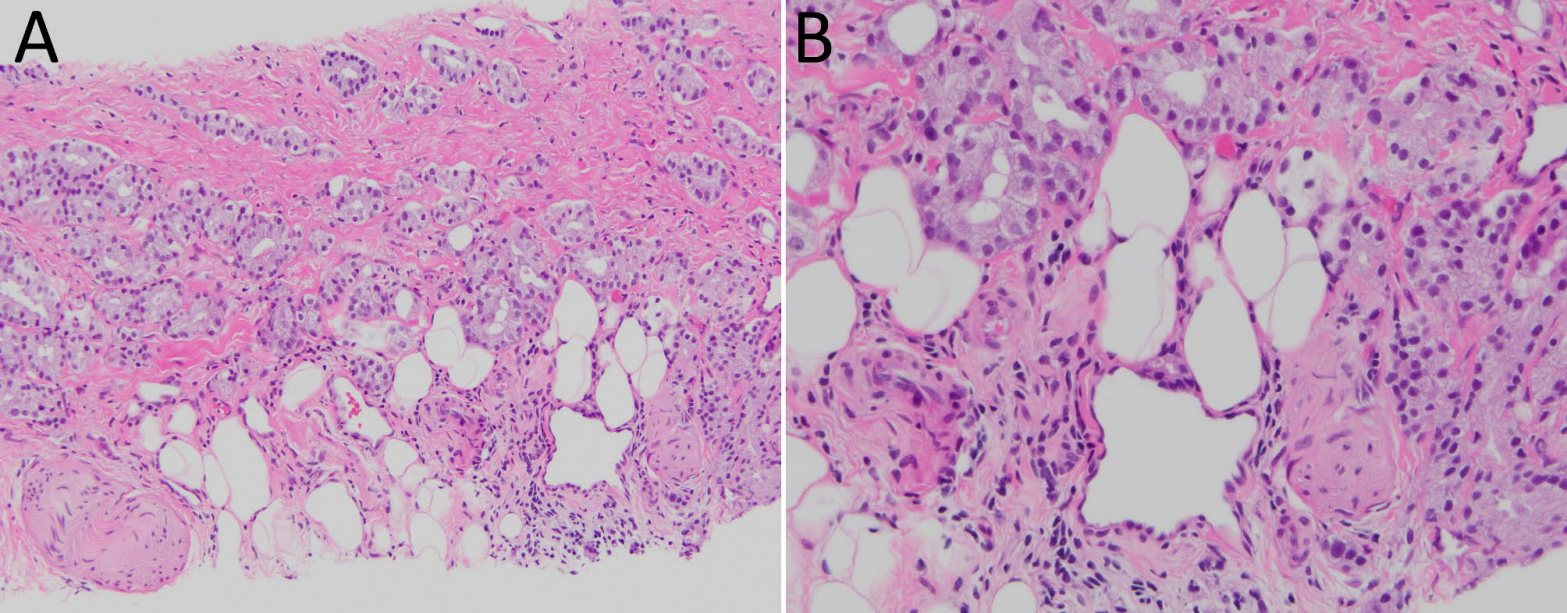
Extraprostatic extension. **A-B** Medium and high-power views depict tumor infiltration to periprostatic adipose tissue. Perineural invasion is also seen (H&E stain, 200X & 400X magnification, respectively)

This study was approved by the institutional review board of our hospital. We retrospectively reviewed patients with PCa who had undergone concomitant SBx and TBx using the TR (12-core SBx, n = 1917) or TP (20-core SBx, n = 931) approach in our institution between January 2015 and July 2022. Clinicopathological factors including age, prebiopsy prostate-specific antigen (PSA), prostate volume, indication for biopsy, index MRI target lesion size, number of reported mpMRI targets, Prostate Imaging Reporting & Data System (PI-RADS) score, locations of index MRI targets, and pathology results of obtained biopsy cores were collected. All prostate biopsies were reviewed by two genitourinary pathologists (CLW and HWC). In each case, the overall Gleason scores for SBx and TBx were based on the core with the highest score. According to the recommendations of the International Society of Urological Pathology [11] and the Genitourinary Pathology Society [12], the Gleason Grade Group (GG) was assigned to the biopsy cores. EPE on biopsy was defined as the presence of cancer cells within or immediately adjacent to periprostatic adipose tissue (Fig. 1) [13].

### Transperineal and transrectal prostate biopsy

**Fig. 2:**
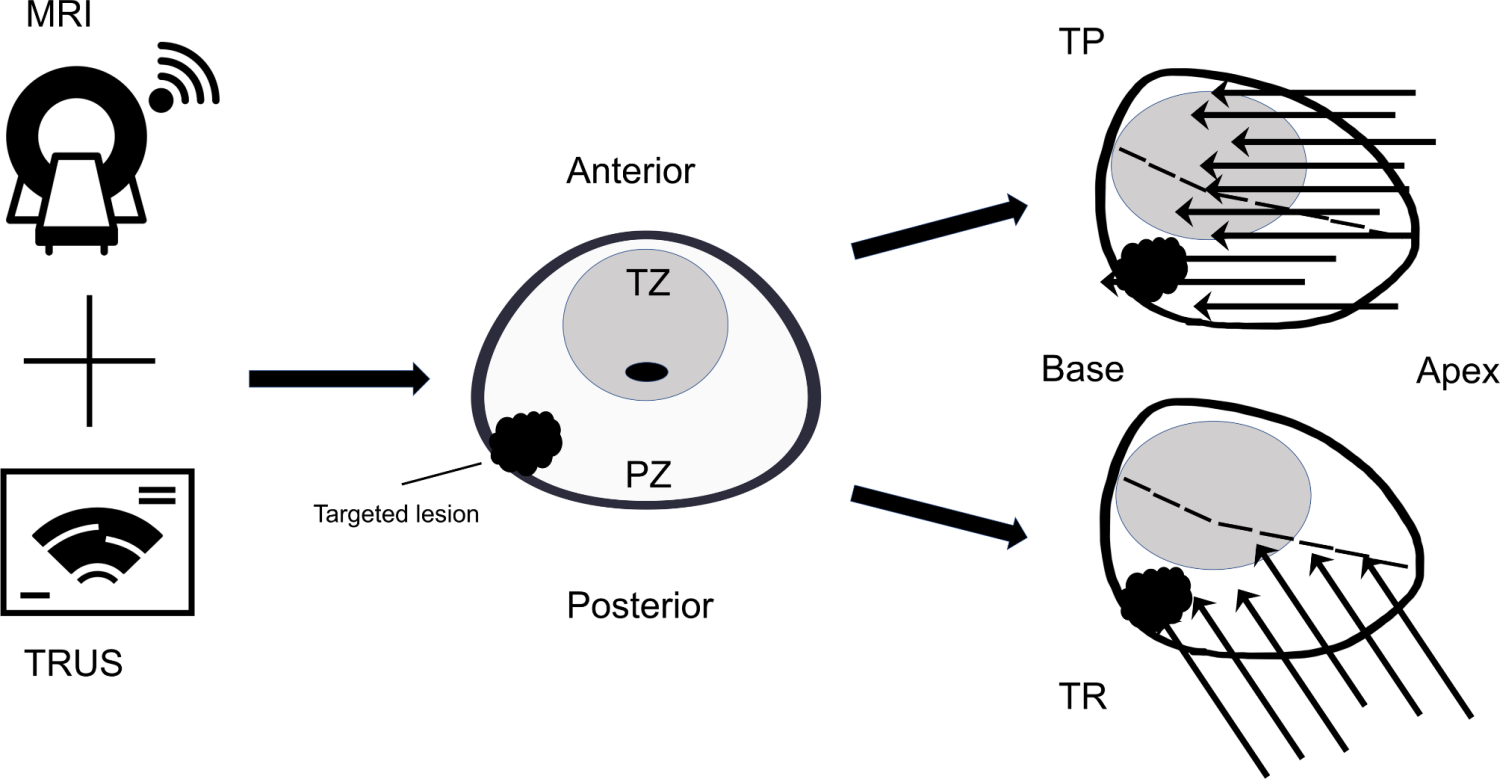
Workflow for TP and TR multiparametric MRI-US fusion targeted combined with systemic template prostate biopsy. Multiparametric MRI images are acquired to identify the prostate and lesions, fusing with real-time US to identify specific areas for biopsy. All patients underwent 3–4 targeted biopsy cores followed by concomitant systematic template biopsies. Systematic biopsy cores in the TP approach are obtained using 2 cores (different locations) taken from each of the 10 sites bilaterally (5 sides each). For the TR approach, a standard 12-core biopsy in a double sextant template is performed. Abbreviations: TP, Transperineal; TR, Transrectal; MRI, Magnetic resonance imaging; US, Ultrasound

All patients had one or more suspicious lesions identified on the prior prostate mpMRI. MRI-identified regions of interest were assigned using PI-RADS v2.1 [14] scoring. All patients underwent 2–4 fusion-targeted biopsy cores per lesion with software fusion (UroNav MRI-US fusion system Philips, Amsterdam, Netherlands) combined with concomitant SBx through either the TR or TP approach performed by five urologists, respectively, as described in previous studies (Fig. 2) [15–17].

### Statistical analysis

Descriptive statistics for categorical variables focused on frequencies and proportions. The medians and interquartile ranges (IQRs) were reported for continuous variables. Statistical analysis was performed using the Mann–Whitney U test for continuous variables and the 2-sample McNemar test, Pearson’s chi-squared test, or Fisher’s exact test for categorical variables. All tests were two-sided, with statistical significance set at *p* < 0.05. All statistical analyses were performed using the SPSS software (version 20.0; SPSS Inc., Chicago, IL, USA).

## Results

In total, 2848 patients underwent concomitant SBx and TBx using the TR (n = 1917) or TP (n = 931) approaches. Among them, 40 cases (1.4%) were diagnosed with EPE, of which 20 cases were diagnosed using TR (1.0%) and 20 cases were diagnosed using TP (2.1%). The EPE detection rate through TP was significantly higher than that through TR (*p* = 0.019). The significance was maintained for both SBx (TR:0.7% vs. TP:1.6%; *p* = 0.028) and TBx (TR:0.5% vs. TP:1.2%; *p* = 0.033) (Table 1).

**Table 1:** Extraprostatic extension detection rate in patients undergone both systematic template and MRI-US fusion guided prostate biopsy through transrectal or transperineal approach: comparison between transrectal and transperineal approach

Methods	All (n = 2848)	TR (n = 1917)	TP (n = 931)	*p* value
EPE-detection rate, n (%)				
Combined	40 (1.4)	20 (1.0)	20 (2.1)	**0.019**
SBx	29 (1.0)	14 (0.7)	15 (1.6)	**0.028**
TBx	20 (0.7)	9 (0.5)	11 (1.2)	**0.033**

*TR* transrectal fusion guided prostate biopsy, *TP* transperineal fusion guided prostate biopsy, *EPE* extraprostatic extension, *SBx* systemic template biopsy, *TBx* MRI-US fusion targeted biopsy, *Combined* diagnosis by SBx and/or TBx, *p* TR VS TP, *p*-values marked with bold indicate statistically significant differences

The details of the EPE lesions between the TR and TP are shown in Table 2. We found that many more cores in the non-base locations were identified in the TP than in the TR (76.7% vs. 50.0%, *p* = 0.038).

**Table 2:** Comparison of total extraprostatic extension lesions and locations between transrectal and transperineal fusion guided prostate biopsy in 40 prostate cancer cases with identifiable extraprostatic extension

Variables	TR (n = 20)	TP (n = 20)	*p* value
Total EPE#, n	26	30	
Mean EPE#/case	1.3	1.5	
EPE location, n (%)			**0.038**
Base	13 (50.0)	7 (23.3)	
Non-base	13 (50.0)	23 (76.7)	
Laterality, n (%)			0.539
Right	10 (38.5)	13 (43.3)	
Left	15 (57.7)	17 (56.7)	
Midline	1 (3.8)	0 (0)	
EPE in Bx type, n (%)			0.666
SBx	15 (57.7)	19 (63.3)	
TBx	11 (42.3)	11 (36.7)	

*TR* transrectal fusion guided prostate biopsy, *TP* transperineal fusion guided prostate biopsy, *EPE* extraprostatic extension, *SBx* systemic template biopsy, *TBx* MRI-US fusion targeted biopsy, *p*-values marked with bold indicate statistically significant differences.

Of the 40 EPE cases, 20 (50%), 11 (27.5%), and 9 (22.5%) were identified only in SBx, only in TBx, and both in SBx and TBx, respectively. SBx showed a higher EPE detection rate than TBx in both the individual approach and the combination but did not reach statistical significance (combined: *p* = 0.196; TR: *p* = 0.296; TP: *p* = 0.430) (Table 3).

**Table 3:** Comparison of extraprostatic extension between systematic template biopsy and fusion guided prostate biopsy

Biopsy methods	All (n = 2848)	TR (n = 1917)	TP (n = 931)
EPE-detection rate, n (%)			
Combined methods	40 (1.4)	20 (1.0)	20 (2.1)
EPE detected in SBx	29 (1.0)	14 (0.7)	15 (1.6)
In SBx only	20 (0.7)	11 (0.6)	9 (1.0)
In both SBx and TBx	9 (0.3)	3 (0.2)	6 (0.6)
EPE detected in TBx	20 (0.7)	9 (0.5)	11 (1.2)
In TBx only	11 (0.4)	6 (0.3)	5 (0.5)
In both SBx and TBx	9 (0.3)	3 (0.2)	6 (0.6)
*p* value	0.196	0.296	0.430

*TR* transrectal fusion guided prostate biopsy, *TP* transperineal fusion guided prostate biopsy, *EPE* extraprostatic extension, *SBx* systemic template biopsy, *TBx* MRI-US fusion targeted biopsy, *Combined* diagnosis by SBx and/or TBx, *p* EPE detected in SBx vs. EPE detected in TBx

A comparison of SBx and TBx is presented in Supplementary Table 1. All variables in both groups showed no significant differences, except for higher PCa-positive cores in SBx than in TBx (10 vs. 4, *p* < 0.001) and higher PCa-positive core rate in TBx than in SBx (100% vs. 73.0%, *p* < 0.001).

The clinicopathological characteristics of all the EPE-positive patients are shown in Table 4. The median age at biopsy was 71 years (IQR, 64–76), the median prebiopsy PSA was 11.9 ng/mL (IQR, 6.1–17.2), the median prostate volume from MRI examination was 52 cc (IQR, 38–66), and the prebiopsy PSA density (PSAD) was 0.25 ng/mL/cc (IQR, 0.14–0.44). In addition, TP showed a higher PSAD (0.35 vs. 0.17, *p* = 0.005), a greater number of GG4–5 in the core with EPE (65.0% vs. 50.0%, *p* = 0.020), and more PCa-positive SBx cores (10 vs. 8, *p* = 0.023) than in TR.

**Table 4:** Baseline clinico-radiological-pathological characteristics in 40 prostate cancer cases who performing both systematic template and MRI-US fusion guided prostate biopsy with identifiable extraprostatic extension through transrectal or transperineal approach

Variables	All	TR	TP	*p* value
Patients, n (%)	40 (100)	20 (50.0)	20 (50.0)	
Median yrs age (IQR)	71 (64–76)	72 (66–78)	73 (63–75)	0.904
Median ng/ml PSA (IQR)	11.9 (6.1–17.2)	9.53 (5.8–13.9)	14.5 (6.8–30.9)	0.056
Median cc prostate vol (IQR)	52 (38–66)	52 (39–70)	52 (34–65)	0.620
Median ng/ml/cc PSA density (IQR)	0.25 (0.14–0.44)	0.17 (0.12–0.25)	0.35 (0.24–0.71)	**0.005**
Indication for biopsy, n (%)				0.223
Elevated PSA, no prior biopsy	31 (77.5)	15 (75.0)	16 (80.0)	
PCa + active surveillance	6 (15.0)	2 (10.0)	4 (20.0)	
Elevated PSA + prior negative biopsy	3 (7.5)	3 (15.0)	0 (0)	
Median index diameter (cm)	2.3 (1.5–3.5)	2.3 (1.5–3.4)	2.5 (1.5–4.1)	0.445
No. targets on MRI, n (%)				0.451
1	31 (77.5)	17 (85.0)	14 (70.0)	
>1	9 (22.5)	3 (15.0)	6 (30.0)	
PI-RADS score (index), n (%)				1.000
3	2 (5.0)	1 (5.0)	1 (5)	
4	8 (20.0)	4 (20.0)	4 (20.0)	
5	30 (75.0)	15 (75.0)	11 (75.0)	
Primary location (index) on MRI, n (%)				0.407
Transitional zone	1 (2.5)	1 (5.0)	0 (0)	
Peripheral zone	32 (80.0)	17 (85.0)	15 (75.0)	
Multiple zones	7 (17.5)	2 (10.0)	5 (25.0)	
Secondary location (index) on MRI, n (%)				0.458
Posterior	25 (62.5)	14 (70.0)	11 (55.0)	
Anterior	3 (7.5)	2 (10.0)	1 (5.0)	
Both	12 (30.0)	4 (20.0)	8 (40.0)	
Laterality (index), n (%)				0.668
Right	12 (30.0)	5 (25.0)	7 (35.0)	
Left	18 (45.0)	9 (45.0)	9 (45.0)	
Both	10 (25.0)	6 (30.0)	4 (20.0)	
EPE suspected on MRI, n (%)				1.000
Present	18 (45.0)	11 (55.0)	11 (55.0)	
Absent	22 (55.0)	9 (45.0)	9 (45.0)	
Total Gleason Grade Group, n (%)				0.630
2	6 (15.0)	2 (10.0)	4 (20.0)	
3	5 (12.5)	3 (15.0)	2 (10.0)	
4	11 (27.5)	7 (35.0)	4 (20.0)	
5	18 (45.0)	8 (40.0)	10 (50.0)	
Gleason Grade Group in EPE, n (%)				**0.020**
1	1 (2.5)	1 (5.0)	0 (0)	
2	6 (15.0)	1 (5.0)	5 (25.0)	
3	10 (25.0)	8 (40.0)	2 (10.0)	
4	11 (27.5)	7 (35.0)	4 (20.0)	
5	12 (30.0)	3 (15.0)	9 (45.0)	
PNI (Combined), n (%)				1.000
Present	39 (97.5)	19 (95.0)	20 (100)	
Absent	1 (2.5)	1 (5.0)	0 (0)	
PNI in EPE				1.000
Present	36 (90.0)	18 (90.0)	18 (90.0)	
Absent	4 (10.0)	2 (10.0)	2 (10.0)	
Median PCa-positive cores (IQR)				
SBx	10 (5–13)	8 (3–11)	10 (8–15)	**0.023**
TBx	4 (3–5)	4 (3–5)	4 (3–5)	0.904
Median PCa-positive core rate (IQR)				
SBx	0.73 (0.35–0.94)	0.63 (0.29–0.98)	0.73 (0.41–0.94)	0.968
TBx	1.00 (0.88-1.00)	1.00 (0.81-1.00)	1.00 (1.00–1.00)	0.565
Median GPC (IQR)				
SBx	0.95 (0.80-1.00)	0.95 (0.75-1.00)	0.95 (0.80-1.00)	0.947
TBx	0.95 (0.85-1.00)	0.95 (0.74-1.00)	0.95 (0.86-1.00)	0.904
Median GPC with EPE (IQR)				
SBx	0.90 (0.64–0.98)	0.90 (0.48–0.95)	0.95 (0.80-1.00)	0.310
TBx	0.95 (0.85–0.99)	0.95 (0.70-1.00)	0.95 (0.85–0.95)	0.941

*TR* transrectal fusion guided prostate biopsy, *TP* transperineal fusion guided prostate biopsy, *PSA* prostate-specific antigen, *PCa* prostate cancer, *EPE* extraprostatic extension, *SBx* systemic template biopsy, *TBx* MRI-US fusion targeted biopsy, *Combined* diagnosis by SBx and/or TBx, *PNI* perineural invasion, *GPC* greatest percentage of cancer involvement, *p* TR VS TP, *p*-values marked with bold indicate statistically significant differences

A comparison of patients with or without an EPE diagnosis on MRI is shown in Supplementary Table 2. There were no significant differences between the two groups, except for patients diagnosed with EPE on MRI, showing a higher index diameter (2.7 cm vs. 2.0 cm, *p* = 0.045) and higher frequency of a PI-RADS score of 5 (90.9% vs. 55.6%, *p* = 0.021) compared to those without.

Among 40 biopsy EPE cases, 12 (30.0%) underwent subsequent RP, and EPE was identified in all RP specimens (Supplementary Table 3). Furthermore, 83.3% (10 out of 12 cases) of biopsy EPE locations were consistent with the MRI index tumor location, and 100% of biopsy EPE locations were consistent with the EPE locations identified in the RP specimens. Furthermore, seven cases (58.3%) had a tumor volume ≥ 30% of the prostate, and four (33.3%) had a final GG5 in the RP specimen. All 12 cases (100%) showed perineural invasion (PNI) in both biopsy and RP specimens, while five cases (41.7%) had a positive surgical margin and two cases (16.7%) had seminal vesicle invasion.

Excluding the 12 patients who received RP, 26 (92.9%) of the remaining 28 patients received radiation (19 cases) or androgen deprivation therapy (7 cases). In a median follow-up period of 22 months (IQR, 6–49 months) after treatment, four patients developed BCR. Of the 40 patients, 14 (35%) developed regional and/or distant metastases. One patient (2.5%) died of PCa during the follow-up period (Supplementary Table 4).

## Discussion

Adenocarcinoma infiltration of adipose tissue in a biopsy is widely recognized as a histological criterion for diagnosing EPE, as intraprostatic adipose tissue is extremely rare [4]. MRI can diagnose EPE; however, its sensitivity and specificity are insufficient and unreliable. An accurate estimate of the preoperative probability of EPE is critical to guide decision-making regarding curative intention management. To the best of our knowledge, this is the first study to compare the detection of EPE and its associated findings when using the TR and TP approaches. Our study found that the TP approach had a significantly higher EPE detection rate than the TR approach for SBx, TBx, or the combined methods. The higher detection frequency of EPE in the TP approach compared to the TR approach in SBx or combined SBx and TBx may be due to the greater number of cores taken during the TP approach (20 cores) compared to the TR approach (12 cores) [18]. However, the significant difference between the two approaches in TBx, using the same cores, indicated that the TP approach could increase the EPE detection rate compared to the TR approach.

The regions of the 20-core TP biopsy scheme used in our study [16] were grouped into 10 areas: right anterior medial, right anterior lateral, right posterior medial, right posterior lateral, right base, left anterior medial, left anterior lateral, left posterior medial, left posterior lateral, and left base, based on laterality (right and left, anterior/posterior, middle/lateral, and base or non-base). Two cores were taken from each region according to the standard template, for a total of 20 cores. For cores taken from other than the right and left bases, each biopsy core typically captures the length of the prostate, including the apex, middle, and base areas. However, for the four cores from the base, the surgeons collect the sample by inserting halfway into the prostate without passing the apex. Previously, in RP specimen studies, the most commonly reported location of EPE was at the posterolateral region of prostate, especially the mid-portion or the base [19]. In TR biopsy studies, EPE was also reported to be found mainly in the posterolateral areas of the prostate [5], and extensive cancer at the base predicted EPE on the biopsy [20]. According to Fleshner et al. [5], in their study of 183 cases using the TR ultrasound-guided biopsy technique, EPE was found at the base of the prostate in 67 (37%) cases. Moreover, in a recent meta-analysis by Tu et al. [21], PCa detection rate by TBx was better using the TP approach than the TR approach, especially in detecting anterior tumors. In our study, 50% of EPE lesions were detected at the base area using the TR approach, which was significantly higher than the 23.3% detected using the TP approach. Furthermore, in accordance with the finding of Fleshner et al. [5] that most patients had one or two cores with EPE, we also found a mean number of cores with EPE of 1.40 (56/40) in all cases, with a slightly higher number in TP cases (1.50, 30/20) than in TR cases (1.30, 26/20). Overall, our findings not only confirm that biopsy EPE is often located at the base but also indicate that the TP biopsy technique increases the likelihood of detecting EPE from all regions of the prostate but may sacrifice the detection rate at the base.

In our study, SBx consistently showed a higher EPE detection rate than TBx for both TR and TP approaches, although the difference was not statistically significant. In contrast to our findings, in a small sample size cohort (n = 333) with only five EPE cases identified, Baumgartner et al. [9] reported that TBx showed significantly better performance in detecting EPE than SBx. In general, TBx is superior to SBx for detecting csPCa [22]. However, Pepe et al. [23] found that SBx with a median 30-core template diagnosed 98.3% (59/60) of csPCa cases, while TR fusion and mpMRI-TRUS TP cognitive-targeted biopsy diagnosed 66.7% (40/60) and 93.3% (56/60), respectively. Owing to the overall rarity and lower extent of EPE, it is possible that SBx will have a higher chance of capturing EPE than TBx because of the higher number of cores. In addition, Fasciano et al. [24] showed that the Targeted-Grossing assessment can only identify 32 cases with EPE, where a total of 39 cases with EPE were present on examination of the total tissue. Combined with our findings, the combination of SBx and TBx may be a better choice for EPE detection than TBx alone.

PNI has been shown to be one of the main mechanisms of PCa extension from the prostatic parenchyma to the periprostatic soft tissue [10]. Multiple studies have shown that PNI on needle biopsy is predictive of EPE in univariate analysis [25]. In our study, 97.5% of the EPE-positive cases and 90.0% of the cores with EPE showed concomitant PNI. This finding confirmed the strong correlation between PNI and EPE and suggested that PNI could be the mechanism for PCa extension.

We further evaluated the characteristics of 12 patients who underwent RP. EPE was identified in all RP specimens, which corroborates previous studies that reported a positive predictive value of 91–96% for EPE on biopsy [2, 4, 5]. Furthermore, among the 12 RP specimens, eight (66.7%) showed multi-EPE locations that indicated extensive non-focal EPE, which was consistent with the higher non-focal EPE rate of 88% in a previous study by Fleshner et al. [5]. Recently, Tolonen et al. [26] and Chen et al. [27] reported a higher biopsy incidence of EPE (4.9% [33/670] and 6.7% [117/1742], respectively) in their studies. Compared with our current study, the study cohort included a significantly higher percentage of patients with distant metastases (45.5% (15/33) and 59.8% (70/117), respectively), but much lower (12.5% (5/40)) in our cohort.

The present study has a number of limitations. First, the retrospective nature of our study might cause selection bias. Second, in our study, EPE cases detected using the TP approach were found to have a higher PSAD and more cases with metastases than those detected using the TR approach. PSAD has been reported to be a strong predictor of adverse pathological features, such as EPE, after RP [28, 29]. Although the findings in our study suggest that the TP approach may be more sensitive than the TR approach in detecting EPE, a higher PSAD and higher portion of cases with metastases may indicate that patients in the TP group may potentially carry more advanced diseases. Third, owing to the rare nature of EPE, the sample sizes were relatively small, particularly because the goal of our study was to compare the differences between TR and TP. Finally, since TP biopsy was relatively new, the duration of follow-up after treatment was short (4 months, IQR:3–11). In the future, large prospective randomized studies comparing the two approaches would be ideal for validating our findings.

## Conclusions

TBx can improve csPCa detection when compared to SBx method, but not in detecting in EPE. For patients with adverse clinical features prior to biopsy, a combination of 20-core template SBx and TBx through the TP approach may improve the chance of detecting EPE.

### Electronic supplementary material

Below is the link to the electronic supplementary material.

Supplementary Material 1

Supplementary Material 2

Supplementary Material 3

Supplementary Material 4

## Data Availability

The data used to support the findings of this study are available from the corresponding author upon request.

## Abbreviations

EPE: Extraprostatic extension
PCa: Prostate cancer
RP: Radical prostatectomy
BCR: Biochemical recurrence
mpMRI: Multiparametric magnetic resonance imaging
US: Ultrasound
TBx: mpMRI-US fusion-targeted biopsy
cs: Clinically significant
SBx: Systematic template biopsy
TP: Transperineal
TR: Transrectal
PSA: Prostate-specific antigen
PI-RADS: Prostate Imaging Reporting and Data System
GG: Gleason Grade Group
IQRs: interquartile ranges
PSAD: PSA density
PNI: Perineural invasion
