# Supplementary material for: Detection of extraprostatic extension by transperineal multiparametric magnetic resonance imaging-ultrasound fusion targeted combined with systemic template prostate biopsy
Source: Diagn Pathol. 2023 Sep 11;18:101. doi: 10.1186/s13000-023-01386-w (PMC10494402; doi:10.1186/s13000-023-01386-w)
Supplement: Supplementary file 1 — Supplementary Material 1 [file 13000_2023_1386_MOESM1_ESM.docx]

**Supplementary Table 1** The comparison between standard template biopsy and MRI-US fusion targeted biopsy

| **Variables** | **SBx (n=40)** | **TBx (n=40)** | ***p* value** | **TR-SBx (n=20)** | **TR-TBx (n=20)** | ***p* value** | **TP-SBx (n=20)** | **TP-TBx (n=20)** | ***p* value** |
| --- | --- | --- | --- | --- | --- | --- | --- | --- | --- |
| GG |  |  | 0.727 |  |  | 1.000 |  |  | 1.000 |
| <4 | 13 (32.5) | 15 (37.5) |  | 7 (35.0) | 8 (40.0) |  | 6 (30.0) | 7 (35.0) |  |
| ≥4 | 27 (67.5) | 25 (62.5) |  | 13 (65.0) | 12 (60.0) |  | 14 (70.0) | 13 (65.0) |  |
| PNI, n (%) |  |  | 1.000 |  |  | 0.500 |  |  | 1.000 |
| Present | 37 (92.5) | 36 (90.0) |  | 19 (95.0) | 17 (85.0) |  | 18 (90.0) | 19 (95.0) |  |
| Absent | 3 (7.5) | 4 (10.0) |  | 1 (5.0) | 13 (15.0) |  | 2 (10.0) | 1 (5.0) |  |
| EPE, n (%) |  |  | 0.151 |  |  | 0.332 |  |  | 0.424 |
| Present | 29 (72.5) | 20 (50.0) |  | 14 (70.0) | 9 (45.0) |  | 15 (75.0) | 11 (55.0) |  |
| Absent | 11 (27.5) | 20 (50.0) |  | 3 (30.0) | 11 (55.0) |  | 5 (25.0) | 9 (45.0) |  |
| Median PCa-positive cores (IQR) | 10 (5-13) | 4 (3-5) | **<0.001** | 8 (3-11) | 4 (3-5) | **0.003** | 10 (8-15) | 4 (3-5) | **<0.001** |
| Median PCa-positive core rate (IQR) | 0.73 (0.35-0.94) | 1.00 (0.88-1.00) | **<0.001** | 0.63 (0.29-0.98) | 1.00 (0.81-1.00) | **0.003** | 0.73 (0.41-0.94) | 1.00 (1.00-1.00) | **<0.001** |
| Median GPC (IQR) | 0.95 (0.80-1.00) | 0.95 (0.85-1.00) | 0.975 | 0.95 (0.75-1.00) | 0.95 (0.74-1.00) | 0.659 | 0.95 (0.80-1.00) | 0.95 (0.86-1.00) | 0.412 |
| Median GPC with EPE (IQR) | 0.90 (0.64-0.98) | 0.95 (0.85-0.99) | 0.379 | 0.90 (0.48-0.95) | 0.95 (0.70-1.00) | 0.305 | 0.95 (0.80-1.00) | 0.95 (0.85-0.95) | 1.000 |

*SBx* systemic template biopsy, *TBx* MRI-US fusion targeted biopsy, *TR* transrectal prostate biopsy, *TP* transperineal prostate biopsy, *GG* Gleason Grade Group, *PNI* perineural invasion, *EPE* extraprostatic extension, *PCa* prostate cancer, *GPC* greatest percentage of cancer involvement, *p*-values marked with bold indicate statistically signiﬁcant differences
