# Supplementary material for: Detection of extraprostatic extension by transperineal multiparametric magnetic resonance imaging-ultrasound fusion targeted combined with systemic template prostate biopsy
Source: Diagn Pathol. 2023 Sep 11;18:101. doi: 10.1186/s13000-023-01386-w (PMC10494402; doi:10.1186/s13000-023-01386-w)
Supplement: Supplementary file 2 — Supplementary Material 2 [file 13000_2023_1386_MOESM2_ESM.docx]

**Supplementary Table 2** Characteristics between cases with or without extraprostatic extension on MRI

| **Variables** | **EPE suspected**  **on MRI** | **EPE not suspected**  **on MRI** | ***p*** |
| --- | --- | --- | --- |
| Patients, n (%) | 22 (55.0) | 18 (45.0) |  |
| Median yrs age (IQR) | 74 (65-77) | 71 (62-75) | 0.443 |
| Median ng/ml PSA (IQR) | 14.0 (9.1-28.1) | 8.6 (5.6-14.2) | 0.037 |
| Median cc prostate vol (IQR) | 56 (39-75) | 47 (35-60) | 0.299 |
| Median ng/ml/cc PSA density (IQR) | 0.27 (0.16-0.50) | 0.24 (0.09-0.37) | 0.209 |
| Median index diameter (cm) | 2.7 (1.7-4.0) | 2.0 (1.3-3.1) | **0.045** |
| No. targets on MRI, n (%) |  |  | 0.253 |
| 1 | 19 (86.4) | 12 (66.7) |  |
| >1 | 3 (13.6) | 6 (33.3) |  |
| PI-RADS score (index), n (%) | |  | **0.021** |
| 3 | 0 (0) | 2 (11.1) |  |
| 4 | 2 (9.1) | 6 (33.3) |  |
| 5 | 20 (90.9) | 10 (55.6) |  |
| Index tumor location on MRI, n (%) |  |  | 0.540 |
| Posterior | 13 (59.1) | 12 (66.7) |  |
| Anterior | 1 (4.5) | 2 (11.1) |  |
| Both | 8 (36.4) | 4 (22.2) |  |
| Total Gleason Grade Group, n (%) |  |  | 0.230 |
| 2 | 1 (4.5) | 5 (27.8) |  |
| 3 | 3 (13.6) | 2 (11.1) |  |
| 4 | 6 (27.3) | 5 (27.8) |  |
| 5 | 12 (54.5) | 6 (33.3) |  |
| Gleason Grade Group in EPE, n (%) |  |  | 0.053 |
| 1 | 1 (4.5) | 0 (0) |  |
| 2 | 1 (4.5) | 5 (27.8) |  |
| 3 | 5 (22.7) | 5 (27.8) |  |
| 4 | 5 (22.7) | 6 (33.3) |  |
| 5 | 10 (45.5) | 2 (11.1) |  |
| PNI(Combined), n (%) |  |  | 1.000 |
| Present | 21 (95.5) | 18 (100) |  |
| Absent | 1 (4.5) | 0 (0) |  |
| PNI in EPE, n (%) |  |  | 0.613 |
| Present | 19 (86.4) | 17 (94.4) |  |
| Absent | 3 (13.6) | 1 (5.6) |  |
| Median PCa-positive cores (IQR) |  |  |  |
| SBx | 10 (8-14) | 7 (4-12) | 0.219 |
| TBx | 4 (3-5) | 4 (3-5) | 0.968 |
| Median PCa-positive core rate (IQR) |  |  |  |
| SBx | 0.78 (0.44-0.96) | 0.48 (0.29-0.92) | 0.163 |
| TBx | 1.00 (1.00-1.00) | 1.00 (0.77-1.00) | 0.352 |
| Median GPC (IQR) |  |  |  |
| SBx | 0.95 (0.90-1.00) | 0.93 (0.59-1.00) | 0.229 |
| TBx | 0.95 (0.89-1.00) | 0.95 (0.78-1.00) | 0.861 |
| Median GPC with EPE (IQR) |  |  |  |
| SBx | 0.90 (0.80-1.00) | 0.90 (0.38-0.96) | 0.533 |
| TBx | 0.95 (0.90-1.00) | 0.90 (0.75-0.98) | 0.370 |

*EPE* extraprostatic extension, *PSA* prostate-specific antigen, *PCa* prostate cancer, *SBx* systemic template

biopsy, *TBx* MRI-US fusion targeted biopsy, *PNI* perineural invasion, *GPC* greatest percentage of cancer

involvement, *p*-values marked with bold indicate statistically signiﬁcant differences
