# Supplementary material for: Detection of extraprostatic extension by transperineal multiparametric magnetic resonance imaging-ultrasound fusion targeted combined with systemic template prostate biopsy
Source: Diagn Pathol. 2023 Sep 11;18:101. doi: 10.1186/s13000-023-01386-w (PMC10494402; doi:10.1186/s13000-023-01386-w)
Supplement: Supplementary file 3 — Supplementary Material 3 [file 13000_2023_1386_MOESM3_ESM.docx]

**Supplementary Table 3** Characteristics of 12 prostate cancer cases with both biopsy and radical prostatectomy

|  | **MRI** | | **Bx** | | | | | | | **RP** | | | | | |  |
| --- | --- | --- | --- | --- | --- | --- | --- | --- | --- | --- | --- | --- | --- | --- | --- | --- |
|  | **EPE** | **Index tumor loc** | **Bx** | **EPE** | **GG** | **PCR** | **GPC** | **EPE loc** | **PNI** | **Tumor%** | **EPE loc** | **PSM loc** | **SVI** | **GG** | **PNI** | **pN** |
| Case 1 | - | L mid to apex; TZ | TR | TBx | 4 | 0.50 | 0.95 | L lateral | + | 45 | (R/L)P | R apex | - | 5 | + | - |
| Case 2 | + | L base to mid; PZ | TP | SBx | 2 | 0.78 | 0.95 | L base | + | 30 | (R/L)P and A | (R/L)P | - | 2 | + | - |
| Case 3 | - | R PL apex to base; PZ | TP | TBx | 4 | 0.65 | 1.00 | R apex | + | 15 | (R/L)P | (R/L)P | - | 5 | + | - |
| Case 4 | - | L lateral and PL; PZ | TP | SBx | 5 | 0.50 | 1.00 | L AM | + | 20 | LP to LA | Negative | - | 5 | + | - |
| Case 5 | - | L; PZ | TR | SBx | 5 | 0.29 | 0.50 | L base | + | 5 | LP | Negative | - | 5 | + | - |
| Case 6 | - | R mid PL; PZ | TR | SBx | 4 | 0.39 | 0.40 | R apex /mid | + | 5 | RP | Negative | - | 2 | + | - |
| Case 7 | - | R/ L mid to apex; PZ | TR | SBx/TBx | 4 | 0.93 | 1.00 | R base | + | 45 | RA and (R/L)P | RP, (R/L)apex | - | 3 | + | - |
| Case 8 | - | L base; PZ | TR | SBx | 2 | 0.13 | 0.70 | R apex | + | 30 | RP | Negative | - | 2 | + | - |
| Case 9 | + | LP mid; PZ | TR | TBx | 3 | 0.81 | 0.90 | L mid | + | 10 | LP | Negative | - | 3 | + | - |
| Case 10 | + | RP base; PZ | TR | TBx | 3 | 0.62 | 0.95 | R base | + | 30 | (R/L)P | LP | + | 3 | + | - |
| Case 11 | - | RA apex to base; PZ/TZ | TP | TBx | 4 | 0.42 | 0.95 | R apex | + | 30 | (R/L)A | Negative | - | 3 | + | - |
| Case 12 | + | R mid; PZ | TP | SBx | 5 | 1.00 | 1.00 | L base | + | 80 | (R/L)P | Negative | + | 5 | + | - |

*EPE* extraprostatic extension, *Loc* location, *Bx* prostate biopsy, *GG* Gleason Grade Group, *PCR* prostate cancer-positive core rate, *GPC* greatest percentage of cancer involvement, *PNI* perineural invasion, *RP* radical prostatectomy, *PSM* positive surgical margin, *SVI* seminal vesicle invasion, *-* absent, *L* left, *TZ* transitional zone, *TR* transrectal fusion guided prostate biopsy, *TBx* MRI-US fusion targeted biopsy, *+* present, *R* right, *P* posterior, *PZ* peripheral zone, *TP* transperineal fusion guided prostate biopsy, *SBx* systemic template biopsy, *PL* posterior lateral, *A* anterior, *AM* anterior medial
