# Supplementary material for: Detection of extraprostatic extension by transperineal multiparametric magnetic resonance imaging-ultrasound fusion targeted combined with systemic template prostate biopsy
Source: Diagn Pathol. 2023 Sep 11;18:101. doi: 10.1186/s13000-023-01386-w (PMC10494402; doi:10.1186/s13000-023-01386-w)
Supplement: Supplementary file 4 — Supplementary Material 4 [file 13000_2023_1386_MOESM4_ESM.docx]

**Supplementary Table 4** Treatments and Follow-up outcomes of the EPE cases

| **Variables** | **Total (n=40)** | **TR (n=20)** | **TP (n=20)** |
| --- | --- | --- | --- |
| Treatments |  |  |  |
| RP, n (%) | 12 (30.0) | 6 (30.0) | 6 (30.0) |
| RT, n (%) | 19 (47.5) | 11 (55.0) | 8 (40.0) |
| ADT, n (%) | 7 (17.5) | 2 (10.0) | 5 (25.0) |
| ND, n (%) | 3 (7.5) | 1 (5.0) | 2 (10.0) |
| Median follow-up duration after RP or RT (IQR) | 22 (6-49) | 41 (27-62) | 4 (3-11) |
| Outcomes |  |  |  |
| BCR, n (%) |  |  |  |
| Post-RP (n=12) | 2 (2/12, 16.7) | 1 (1/6, 16.7) | 1 (1/6, 16.7) |
| Post-RT (n=19) | 2 (2/19, 10.5) | 2 (2/11, 18.2) | 0 (0) |
| Metastasis, n (%) |  |  |  |
| Lymph node, n (%) | 9 (22.5) | 2 (10.0) | 7 (35.0) |
| Pre-Biopsy | 8 (20.0) | 2 (10.0) | 6 (30.0) |
| Progression after Treatment | 1 (2.5) | 0 (0) | 1 (2.5) |
| Bone, n (%) | 5 (12.5) | 1 (5.0) | 4 (20.0) |
| Pre-Biopsy | 3 (7.5) | 0 (0) | 3 (5.0) |
| Progression after Treatment | 2 (5.0) | 1 (5.0) | 1 (5.0) |
| Death, n (%) | 1 (2.5) | 1 (5.0) | 0 (0) |

*RP* radical prostatectomy, *RT* radiotherapy, *ADT* androgen deprivation therapy,

*ND* Not determined, *BCR* biochemical recurrence
